# Aldehyde dehydrogenase 2 activation in aged heart improves the autophagy by reducing the carbonyl modification on SIRT1

**DOI:** 10.18632/oncotarget.6814

**Published:** 2016-01-03

**Authors:** Bing Wu, Lu Yu, Yishi Wang, Hongtao Wang, Chen Li, Yue Yin, Jingrun Yang, Zhifa Wang, Qiangsun Zheng, Heng Ma

**Affiliations:** ^1^ Department of Cardiology, Tangdu Hospital, Fourth Military Medical University, Xi'an, China; ^2^ Department of Pathology, Xijing Hospital, Fourth Military Medical University, Xi'an, China; ^3^ Department of Physiology, School of Basic medicine, Fourth Military Medical University, Xi'an, China; ^4^ Department of Pathophysiology, School of Basic medicine, Fourth Military Medical University, Xi'an, China

**Keywords:** aging, ALDH2, autophagy, carbonyl stress, SIRT1, Gerotarget

## Abstract

Cardiac aging is characterized by accumulation of damaged proteins and decline of autophagic efficiency. Here, by forestalling SIRT1 carbonylated inactivation in aged heart, we determined the benefits of activation of aldehyde dehydrogenase 2 (ALDH2) on the autophagy. In this study, the ALDH2 KO mice progressively developed age-related heart dysfunction and showed reduction in the life span, which strongly suggests that ALDH2 ablation leads to cardiac aging. What's more, aged hearts displayed a significant decrease ALDH2 activity, resulting in accumulation of 4-HNE-protein adducts and protein carbonyls, impairment in the autophagy flux, and, consequently, deteriorated cardiac function after starvation. Sustained Alda-1 (selective ALDH2 activator) treatment increased cardiac ALDH2 activity and abrogated these effects. Using SIRT1 deficient heterozygous (Sirt1+/−) mice, we found that SIRT1 was necessary for ALDH2 activation-induced autophagy. We further demonstrated that ALDH2 activation attenuated SIRT1 carbonylation and improved SIRT1 activity, thereby increasing the deacetylation of nuclear LC3 and FoxO1. Sequentially, ALDH2 enhanced SIRT1 regulates LC3-Atg7 interaction and FoxO1 increased Rab7 expression, which were both necessary and sufficient for restoring autophagy flux. These results highlight that both accumulation of proteotoxic carbonyl stress linkage with autophagy decline contribute to heart senescence. ALDH2 activation is adequate to improve the autophagy flux by reducing the carbonyl modification on SIRT1, which in turn plays an important role in maintaining cardiac health during aging.

## INTRODUCTION

Tight regulation of proteostasis is essential for maintaining cellular homeostasis in postmitotic tissues. Cardiac aging is an intrinsic process that results in impaired cardiomyocyte proteostasis [1]. These degenerative changes are intimately associated with abnormal protein aggregation and impaired protein degradation pathways. Failure to maintain the critical balance between accumulation and clearance may be directly attributed to cardiomyopathies as a consequence of aging [2]. Direct interventions to modulate protein quality control mechanisms may improve health and potentially increase lifespan.

Aldehyde dehydrogenase 2 (ALDH2), an abundantly expressed protein in heart and brain, plays a pivotal role in aldehyde detoxification [3]. Numerous lines of evidence suggest that ALDH2 dysfunction is associated with the process of aging and contribute to age-related cardiovascular diseases [4]. Our previously evidence has revealed that increased myocardium aldehydic load induces covalent carbonyl modification of protein by ‘carbonyl stress’, which results in excessive accumulation of proteoxicity and contributes to heart senescence [5, 6]. Activation of ALDH2 protects the heart against extravagant aging-related myocardial aldehydic load induced proteoxicity. However, a role of ALDH2 in clearance of damaged proteins in aged heart has not been clearly defined.

Along with the ubiquitin proteasome system (UPS), macroautophagy (termed hereafter as autophagy) is important for maintaining proteostasis in the heart, which is upregulated in response to several stressors [7]. In context of quality control, autophagy is responsible for the remove of damaged proteins and abnormal organelles, both of which are hallmarks of aged and dysfunctional tissues [1, 8]. Unfortunately, autophagy/lysosome system exhibits reduced efficiency during cardiac aging [9]. Decreased autophagy is involved in the aging related loss of cardioprotection. Therefore, consideration must be given to both the prevention of carbonyl stress proteotoxicity and the promotion of autophagic function, which may slow down, at least to some extent, the aging-associated damage in heart. We set out to determine the role of ALDH2 in the regulation of autophagy. Our data recapitulated the association between ALDH2 activity and aging-associated autophagy decline and revealed its underlying dysfunctional signaling mechanisms. Specific pharmacological activation of ALDH2 improves the autophagy in aged hearts by reducing the toxic effects of carbonyl stress on the key regulators of autophagy.

## RESULTS

### Reduced ALDH2 activity and increased aldehydic load in aged heart

**Figure 1:**
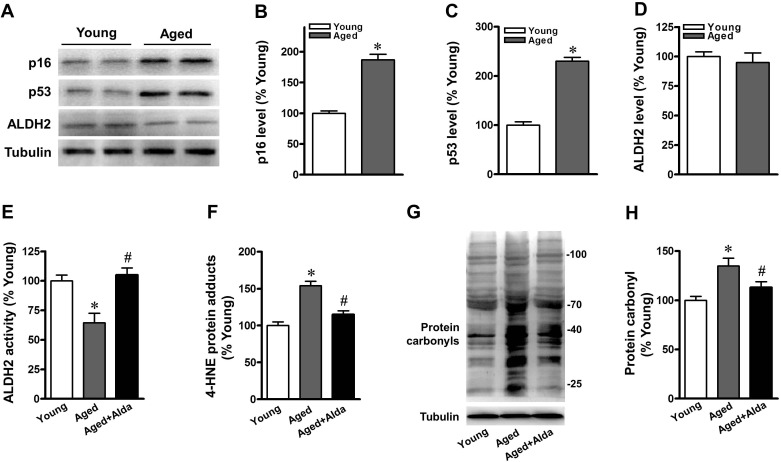
Aged mice show reduced cardiac ALDH2 activity and increased protein carbonyls

Echocardiographic studies were performed to examine hearts with respect to *in vivo* LV function. The heart rate (HR) and percent fractional shortening were similar and in the normal range for both young and aged mice under a basal physiological state (Supplement Table 1). The myocardial senescence marker, ALDH2 protein expression and activity in young and aged C57BL/6 mice were assayed. Expression of p16 and p53, markers of senescence, were significantly increased in the aged heart (Figure 1A-1C). Consistent with our previous findings, aged heart exhibited a declining trend in ALDH2 protein expression but with no significant difference (Figure 1D). However, myocardial ALDH2 activity decreased in aged hearts compared with that in their younger counterparts (Figure 1E). ALDH2 plays a key role in protecting the heart mainly through detoxification of reactive aldehydes, such as 4-hydroxynonenal (4-HNE), and prevents the production of aldehydic adducts [3]. We therefore monitored the effects of selective ALDH2 activation on 4-HNE-protein adducts and total protein carbonyls in aged heart. Aged mice displayed a significant increase of cardiac 4-HNE-protein adducts (Figure 1F) and protein carbonyls (Figure 1G, 1H) compared with relative young controls. We delivered Alda-1 (selective ALDH2 activator) in aged mice. Alda-1 treatment improved cardiac ALDH2 activity by 1.7-fold (Figure 1E) and significantly reduced 4-HNE-protein adducts and protein carbonyls compared with untreated aged hearts (Figure 1F-1H).

**A.** Representative gel blots depicting relative levels of **B.** p16, **C.** p53 and **D.** ALDH2 protein expression in young and aged hearts. The low panel **E.**-**H.** show aged mice with Alda-1(ALDH2 activator) treated, young mice were used as control; **E.** ALDH2 activity; **F.** 4-HNE protein adduct; **G.**, **H.** protein carbonyl formation were assessed by quantificational detection (*n* = 8 per group. *P < 0.05 *vs*. young control, ^#^*P* < 0.05 *vs*. aged group).

### Accelerated aging process in ALDH2 null hearts

**Figure 2:**
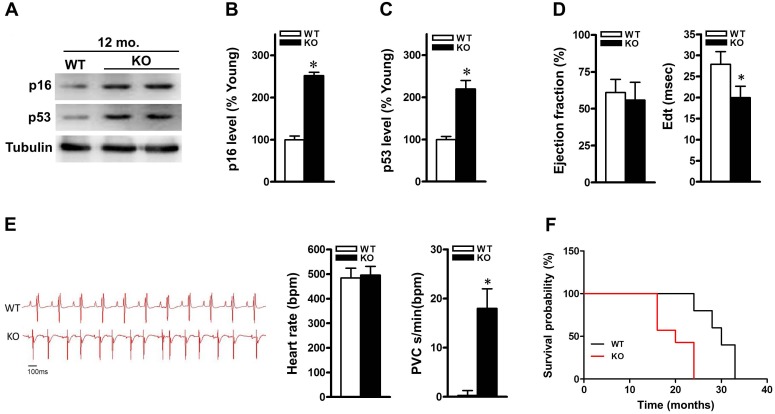
ALDH2 knockout mice display senescent-like features and show impaired heart function

We have demonstrated that ALDH2 activity declines with aging in hearts. To address the role of ALDH2 in the regulation of cardiomyocyte aging process and function, we monitored the cardiac phenotype of ALDH2 KO mice onward until death and compared it with that of age- and sex-matched C57BL/6J WT controls. ALDH2 KO mice demonstrated a normal baseline cardiac phenotype with respect to LV size and function (Supplement Table 2). At 6 and 9 months old, both cardiac morphology and size were unchanged in ALDH2 KO mice compared with that in WT controls. At 12-month-old, however, hearts of ALDH2 KO mice appeared overtly higher aging protein markers (p16 and p53) compared with that in WT controls (Figure 2A-2C). Echocardiography demonstrated that 12-month-old ALDH2 KO hearts were unchanged in systolic function (ejection fraction). Echo-Doppler measurements showed that, however, ALDH2 KO mice had significant impairment in diastolic relaxation at 12 months of age, as demonstrated by a reduction of the E wave deceleration time in the mitral valve inflow pattern measurements (E wave deceleration time) (Figure 2D). ALDH2 KO mice had unchanged heart rhythm compared with those of controls but showed frequent ventricular ectopies, which indicate increased arrhythmia vulnerability (Figure 2E). Our findings indicate that ALDH2 KO mice progressively develop aging process with impaired heart function prior to that of age-matched WT controls. In further support of this, ALDH2 KO mice had shortened life span (Figure 2F), which consistent with previous reports[4]. Altogether, these results suggest that ALDH2 dysfunction is associated with the process of cardiac aging.

**A.** Representative gel blots depicting relative levels of **B.** p16, **C.** p53 protein expression in 12-month-old control and ALDH2 KO hearts. **D.** Quantification of ejection fraction, and E wave deceleration time (EdT) in 12-month-old control and ALDH2 KO mice. **E.** ECG recording in WT control (top) and ALDH2 KO (bottom) mice. Quantification of heart rate (bpm) and ventricular ectopies per minute were assessed in WT control and ALDH2 KO mice. PVC, premature ventricular complexes (*n* = 6 per group, *P < 0.01 *vs*. WT control). **F.** Survival probability of control (black line) and ALDH2 KO (red line) mice, evaluated by using Kaplan-Meier method. Comparison of control and ALDH2 KO survival curves was performed by both Mantel-Cox and Gehan-Breslow-Wilcoxon tests. ALDH2 KO mice show a significant reduction in the life span, as compared with that of controls (*n* = 10, *P* < 0.05).

### ALDH2 ablation leads to impairment in the autophagy

**Figure 3:**
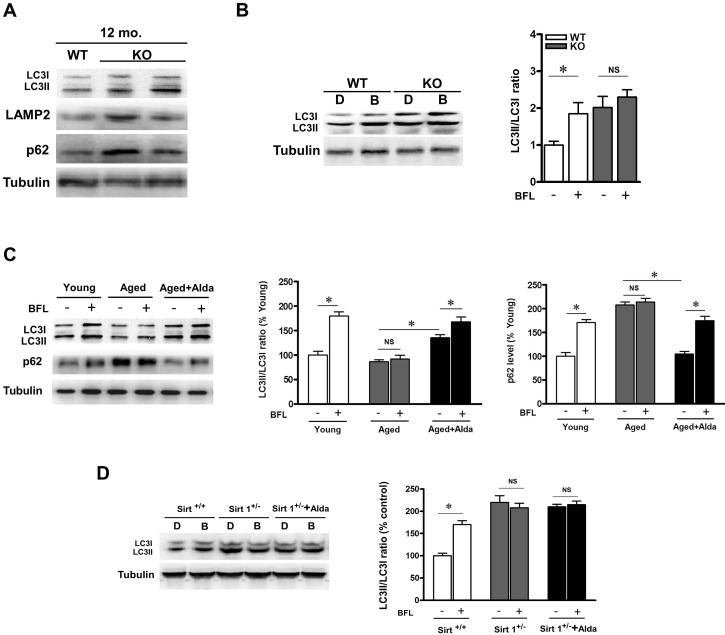
ALDH2 ablation leads to autophagic flux impairment

Autophagy is responsible for the clearance of damaged proteins. The presence of aging-associated protein carbonyl damage prompted us to investigate whether increased autophagy function was detectable. We assessed the state of the autophagy/lysosome system in young, aged and ALDH2 KO hearts. As compared with WT control, 12-month-old ALDH2 KO hearts showed increased levels of lipidated LC3 proteins, LAMP2 and p62 (Figure 3A). We treated WT control and ALDH2 KO mice with bafilomycin (autophagosome-lysosome fusion inhibitor). Bafilomycin increased the LC3-II-to-LC3-I ratio in WT control mice, while it did not elicit any significant further raise in ALDH2 KO hearts (Figure 3B), a finding consistent with autophagic flux impairment. These results suggest that ALDH2 deficiency causes the block of autophagic flux. To further confirm that role of ALDH2 in mediating autophagy, we treated aged mice with Alda-1. Likewise, aged mice hearts showed higher p62 accumulation compared with young controls. However, the LC3II protein levels and increase in p62 in aged hearts were not further enhanced by bafilomycin treatment, suggesting that autophagy flux was impaired in aged heart. In addition, Alda-1 treatment increased LC3-II-to-LC3-I ratio and decreased p62 accumulation in aged heart compared with the untreated aged hearts. Moreover, in Alda-1 treated aged heart, the increase in LC3II and p62 level were further enhanced by bafilomycin (Figure 3C). Altogether, these results suggest that ALDH2 activation enhances autophagy flux in aged heart.

**A.** Western blot analysis of autophagy-related proteins (LC3, LAMP2 and p62) in heart lysates from WT and ALDH2 KO mice at 12 months of age. **B.** Autophagic flux assessment in heart lysates from age-matched WT and ALDH2 KO mice treated either with DMSO or bafilomycin (BFL). Bars represent Relative expression of LC3II to LC3I ratio in mouse groups. **C.** Autophagic flux assessment in heart lysates from young and aged mice treated with vehicle or Alda-1 in the presence or absence of bafilomycin. Bars represent Relative expression of LC3II to LC3I ratio and p62 in mouse groups. Results represent the means from 4 independent experiments, *P < 0.05. **D.** Autophagic flux assessment in heart lysates from WT control and SIRT1 deficient heterozygous (Sirt1^+/−^) mice treated with vehicle or Alda-1 in the presence or absence of bafilomycin. Bars represent Relative expression of LC3II to LC3I ratio in mouse groups. Results represent the means from 4 independent experiments, *P < 0.05.

Sirtuin 1 (SIRT1), an NAD+-dependent protein deacetylase, has been proved to be an effective protector against age-related cardiovascular diseases. To evaluate whether SIRT1 is required for ALDH2-induced autophagy, SIRT1 deficient heterozygous (Sirt1^+/−^) mice were subjected to Alda-1 treatment. Knockdown of SIRT1 induced LC3II and p62 accumulation in the presence and absence of bafilomycin. Furthermore, in Sirt1^+/−^ hearts, ALDH2 activation-induced stimulation of autophagy was not observed (Figure 3D), indicating that SIRT1 is required for ALDH2 activation induced autophagy.

### ALDH2 activation promotes cardiac function after starvation in aged heart

**Figure 4:**
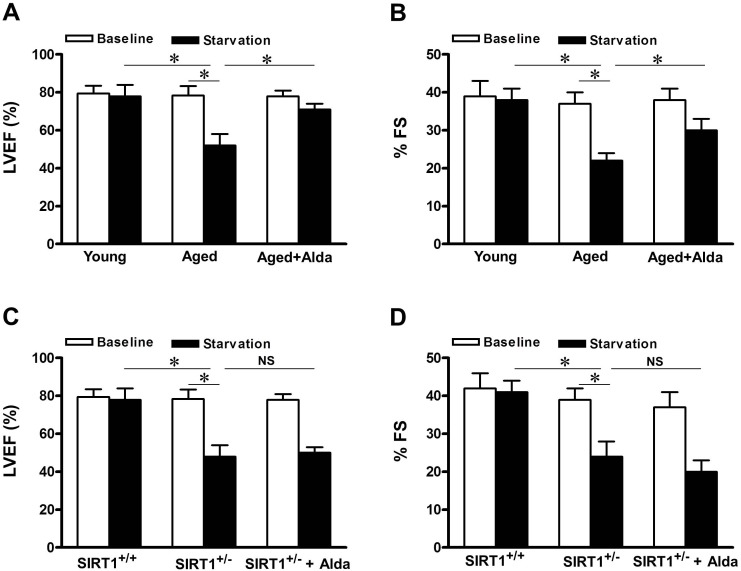
ALDH2 activation improves cardiac function during starvation in aged hearts

Activation of autophagy forms an essential adaptive mechanism for maintaining LV function under starvation conditions [10]. To evaluate the role of ALDH2 enhanced autophagy in regulating cardiac function in aged mice, echocardiographic analyses were conducted before and after starvation. Although cardiac function was maintained in young control mice after 48 hours of food starvation [10], it was significantly deteriorated in aged mice, in which autophagy function is impaired (Figure 3C), consistent with the notion that suppression of autophagy may contribute to aged related cardiac dysfunction. However, in Alda-1 treated aged mice, cardiac function ameliorated significantly after starvation compared with that in untreated aged mice (Figure 4A, 4B). Given that SIRT1 is required for ALDH2-induced autophagy (Figure 3D), SIRT1 deficient heterozygous mice were subjected to starvation. A decrease in cardiac function caused by starvation was also observed in Sirt1^+/−^ mice (Figure 4C, 4D), but, the effect of Alda-1 treatment was negated in Sirt1^+/−^ mice (Figure 4C, 4D). These results suggest that SIRT1 is required for ALDH2 activation-promoted aged heart function after starvation, possibly through stimulation of autophagy.

Cardiac function was evaluated with echocardiographic analyses both at baseline and after 48 hours of starvation. **A.** Evaluations of LV ejection fraction (LVEF) and **B.** fractional shortening (%FS) in young and aged mice treated with vehicle or Alda-1. **C.** LV ejection fraction (LVEF) and **D.** fractional shortening (%FS) in WT and SIRT1 deficient heterozygous (Sirt1^+/−^) mice treated with vehicle or Alda-1. Results represent the means from 4 independent experiments, *P < 0.05.

### ALDH2 activation enhances SIRT1activity in aged heart

**Figure 5:**
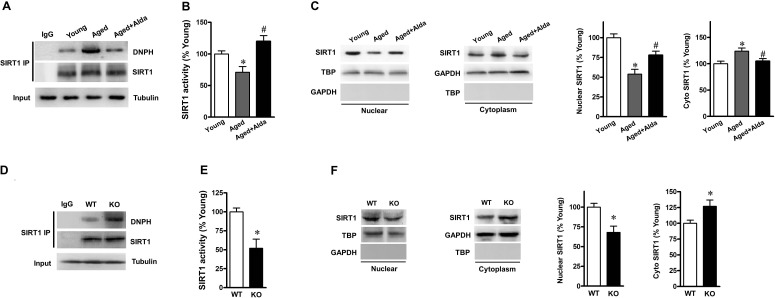
ALDH2 activation enhances SIRT1 activity in aged heart

Given that our results have proved that ALDH2 activation reduces excessive carbonyl stress and enhances autophagic flux in aged hearts; moreover, SIRT1 is required for ALDH2-enhanced autophagy. We hypothesized that ALDH2 prevents SIRT1 inactivation from carbonyl stress, thereby inducing autophagy in aged hearts. In present study, SIRT1 immunoprecipitation and immunoblotting findings indicated that carbonyl modification on SIRT1 was significantly enhanced in aged heart (Figure 5A) and markedly decreased SIRT1 activity (Figure 5B), but Alda-1 treatment significantly reduced the level of carbonylated SIRT1 and restored the SIRT1 activity. Nucleocytoplasmic shuttling played a critical role in regulating SIRT1 activity. To further determine whether ALDH2 activation altered the rate of SIRT1 nuclear-to-cytosolic shuttling in aged heart, nuclear and cytoplasmic SIRT1 were detected in young and aged hearts with or without Alda-1 treatment. As expected, aged heart exhibited decreased levels of nuclear SIRT1, whereas cytoplasmic SIRT1 in aged hearts was higher than those in young groups (Figure 5C). Notably, in aged hearts, Alda-1 treatment resulted in an increase in nuclear SIRT1 and a decrease in cytoplasmic SIRT1 (Figure 5C), both of which indicate that ALDH2 promotes SIRT1 in the aged heart leading to its nuclear localization. Another supporting evidence is that, ALDH2 KO hearts showed a significant increase in carbonylated SIRT1 (Figure 5D) and markedly decreased SIRT1 activity (Figure 5E). Moreover, ALDH2 deficiency elicited a reduction of nuclear SIRT1 but with an increase of cytoplasmic SIRT1 in hearts (Figure 5F). These results suggest that ALDH2 activation could protect aged heart *via* improving cardiac SIRT1 activity by decreasing carbonyl stress and promoting nucleocytoplasmic shuttling of SIRT1.

**A.** Carbonylation of cardiac SIRT1 detected by immunoprecipitation (IP) with SIRT1 antibody followed by western blot with anti-DNPH antibody from young and aged mice treated with vehicle or Alda-1. Anti-tubulin was used as input. **B.** Cardiac SIRT1 activity was assessed by quantificational detection. **C.** Nuclear and cytoplasmic extracts from young and aged hearts were analyzed by immunoblotting. TBP and GAPDH were detected as nuclear and cytoplasmic loading control, respectively. Bar graphs show relative levels of nuclear SIRT1 and cytoplasmic SIRT1 from young and aged mice treated with vehicle or Alda-1 (*n* = 6 per group. *P < 0.05 *vs*. young control, #*P* < 0.05 *vs*. untreated aged group). **D.** WT or ALDH2 KO mice hearts were analyzed for SIRT1 carbonylation with immunoprecipitation and western blot using anti-SIRT1 and anti-DNPH antibodies, respectively. **E.** WT or ALDH2 KO mice were subjected to the SIRT1 activity assay. **F.** Nuclear and cytoplasmic extracts from WT and ALDH2 KO hearts were analyzed by immunoblotting. Bar graphs show relative levels of nuclear SIRT1 and cytoplasmic SIRT1 in mouse groups. (*n* = 6 per group. *P < 0.05 *vs*. WT control).

### ALDH2 activation facilitates deacetylation of LC3 and FoxO1 by SIRT1

**Figure 6:**
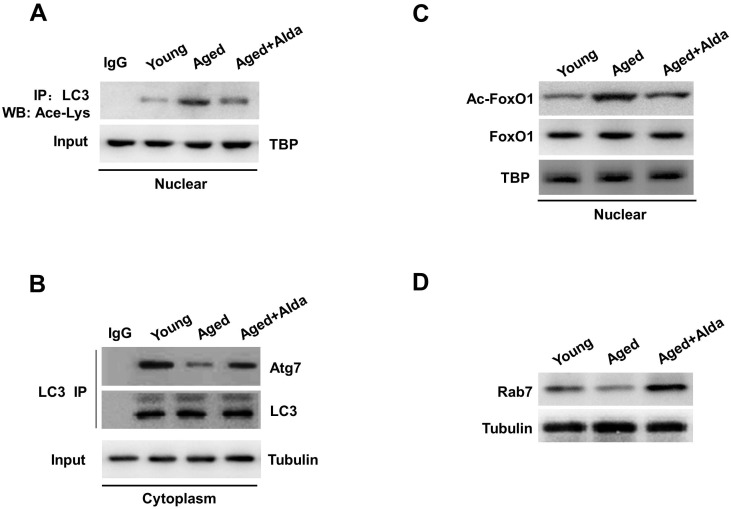
ALDH2 activation facilitates deacetylation of LC3 and FoxO1

Protein acetylation is known to play an important role in autophagy [11]. We next explored the role of SIRT1-dependent deacetylation reactions in regulation of autophagy in aged hearts. Recent *in vitro* study has reported that nuclear localization of LC3 is a prerequisite for this protein to drive autophagy [12], whether nuclear LC3 participates in the ALDH2-induced autophagy in aged heart *in vivo* is unclear. Along with SIRT1 inactivation by carbonylation, we confirmed that, the acetylation of nuclear LC3 was increased in aged hearts compared with young groups, whereas acetylated form of nuclear LC3 was significantly decreased following Alda-1 treatment in aged heart (Figure 6A). Furthermore, autophagosome biogenesis by LC3 requires deacetylated LC3 shift into the cytoplasm and interact with cytoplasmic Atg7 [13]. We found that LC3-Atg7 interaction in cytoplasmic pools was reduced in aged hearts compared with young groups. Of interest, ALDH2 activation by Alda-1treatment markedly enhanced the coimmunoprecipitation of Atg7 with LC3 in aged hearts (Figure 6B). Together, these results suggest that by controlling SIRT1 nuclear localization and its activation, ALDH2 activation in aged hearts augments deacetylation of LC3 in nucleus and subsequent LC3-Atg7 interactions in cytoplasm.

**A.** Nuclear extracts from young and aged mice hearts treated with vehicle or Alda-1 were subjected to immunoprecipitation (IP) with LC3 antibody, and the IP products were further analyzed by immunoblotting for acetyl-lysine. TBP was used as markers for the nuclear fractions. **B.** Cytoplasmic LC3 was immunoprecipitated from young and aged mice hearts treated with vehicle or Alda-1, and the immunocomplexes were subjected to western blot with anti-Atg7 antibody. Tubulin was used as markers for the cytoplasmic fractions. **C.** Acetylation of FoxO1 in nuclear fractions and **D.** immunoblots analyses showing cardiac Rab 7 expression from young and aged mice hearts treated with vehicle or Alda-1.

In addition to autophagosome formation, impairment of autophagic flux can be caused by autophagosome-lysosome fusion. Previous studies have shown that Rab7 is a crucial factor in the maturation of autophagosomes by promoting their fusion with lysosomes, and deacetylation of FoxO1 upregulates the expression of Rab7 in cardiomyocytes [10]. We further examined the role of ALDH2-enhanced SIRT1 activity in deacetylation of FoxO1 and Rab7 expression in aged hearts *in vivo*. An actylated form of FoxO1 in nucleus, evaluated by anti-acetylated FoxO1 antibody, was significantly increased (Figure 6C); conversely, expression of Rab7 was decreased (Figure 6D) in aged hearts. Notably, ALDH2 activation by Alda-1treatment significantly decreased FoxO1 acetylation and enhanced Rab7 expression in aged hearts. Consistent with the block of autophagy flux in ALDH2 KO and aged hearts, these results illustrated that ALDH2 protects SIRT1 activity and deacetylation of LC3 and FoxO1in the nucleus, which in turn controls the major steps in the autophagic flux.

### SIRT1 is essential for ALDH2-ameliorated autophagic flux under carbonyl stress

**Figure 7:**
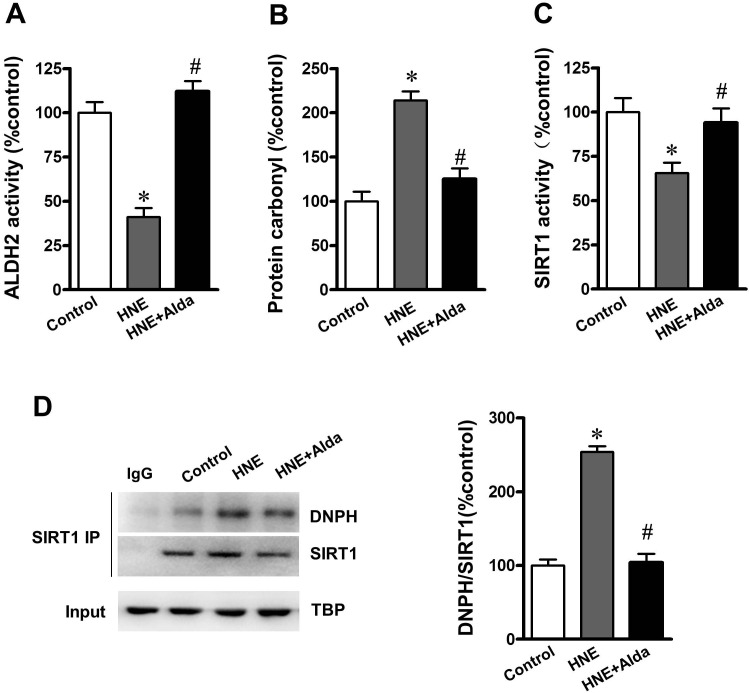
ALDH2 activation reduces SIRT1 carbonylation and improved SIRT1 activity under aldehydic overload

To evaluate whether SIRT1 is required for ALDH2 activation induced autophagy flux under carbonyl stress, cultured cardiomyocytes were exposed to exogenous 4-HNE (2 μmol/L)-induced aldehyde/carbonyl stress with or without ALDH2 activator Alda-1 (20 μmol/L) treatment [14]. It was noted that 4-HNE significantly reduced ALDH2 and SIRT1 activities (Figure 7A-7C). Western bloting and immunoprecipitation indicated that 4-HNE exposure led to increased carbonylated SIRT1, whereas ALDH2 activation significantly reduced the level of carbonylated SIRT1 and restored the SIRT1 activity (Figure 7D).

Cultured cardiomyocytes incubated for 1 hour with vehicle or 40 μmol/L HNE in the absence (control) or presence of 20 μmol/L Alda-1 treatment. **A.** ALDH2 activity, **B.** protein carbonyl formation and **C.** cardiac SIRT1 activity were assessed by quantificational detection. **D.** Representative immunoprecipitation (IP) picture was used to confirm carbonylation of SIRT1. (*n* = 8 per group. *P < 0.05 *vs*. vehicle control, #*P* < 0.05 *vs*. 4-HNE alone).

**Figure 8:**
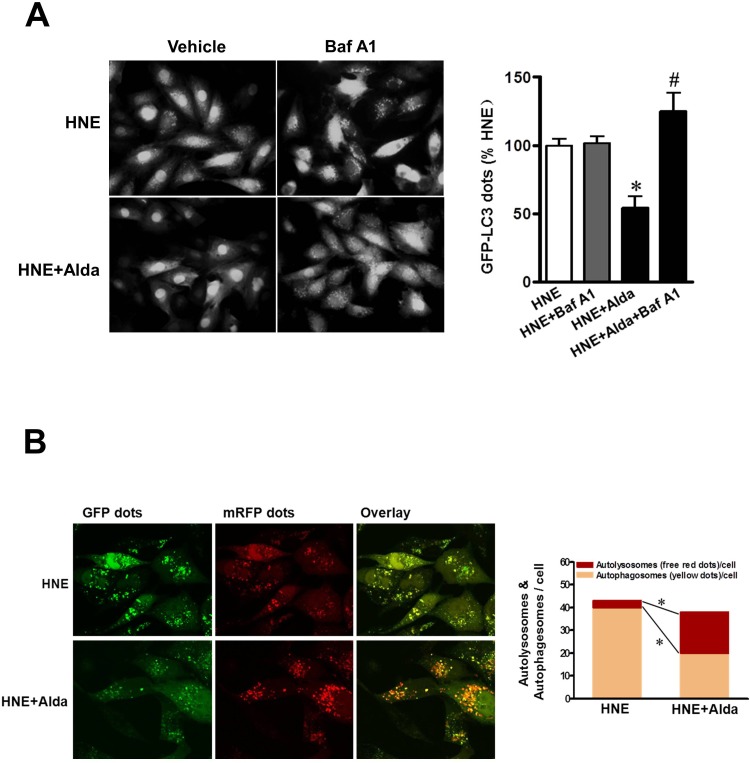
ALDH2 activation ameliorates myocardial autophagic flux under carbonyl stress

Cardiomyocytes were transiently transfected with Ad-GFP-LC3 to label autophagic vesicles, thus enabling quantification of the cell fraction occupied by GFP-LC3 dots. Carbonyl stress by HNE exposure induced GFP-LC3 dots accumulation, and bafilomycin treatment did not increase LC3-positive puncta in 4-HNE exposured cardiomyocytes indicating the block of the autophagic flux under carbonyl stress (Figure 8A). Importantly, Alda-1 treatment was sufficient to decrease the number of LC3-positive puncta and the content was markedly increased following the present of bafilomycin, indicating that accumulated autophagosomes were caused by defective autophagosome removal and ALDH2 activation can induce autophagic flux. Furthermore, to separately evaluate the extent of autophagosome and autolysosome accumulation, we used an adenovirus harboring tandem fluorescent mRFP-GFP-LC3 (Ad-tf-LC3) [10]. The red puncta that overlay with the green puncta and appear yellow in merged images are indicators of autophagosomes, whereas the free red puncta that do not overlay with the green puncta and appear red in merged images are indicative of autolysosomes. Under carbonyl stress by HNE exposure, most of the red puncta overlaid with green puncta, indicating acculmulation of autophagosomes due to block of autophagic flux (Figure 8B). Even in the presence of HNE, Alda-1 treatment restored the autophagic flux by enhanced autophagosome clearance associated with increased autolysosomes formation, as indicated by decrease in yellow puncta in merged images, but not autolysosomes, as indicated by significant increases in free red puncta (Figure 8B). These results validate the notion that ALDH2 activation protects cardiomyocytes from carbonyl stress induced autophgy block.

Cultured cardiomyocytes with or without Alda-1 treatment (20 μmol/L) were incubated with 4-HNE (40 μmol/L) or vehicle in the presence or absence of bafilomycin. **A.** Cardiomyocytes were transfected with Ad-GFP-LC3 for 24 h prior to HNE treatment to label autophagic vesicle and imaged (*n* = 4 per group, *P < 0.05 *vs*. 4-HNE alone; #*P* < 0.05 *vs*. 4-HNE with Alda-1). **B.** Representative images of fluorescent LC3 puncta after mRFP-GFP-LC3 adenovirus transduction (24h). Mean number of autophagosomes represented by yellow puncta in merged images and autolysosomes represented by red puncta in merged images per cell. Results represent the means from 4 independent experiments, *P < 0.05.

**Figure 9:**
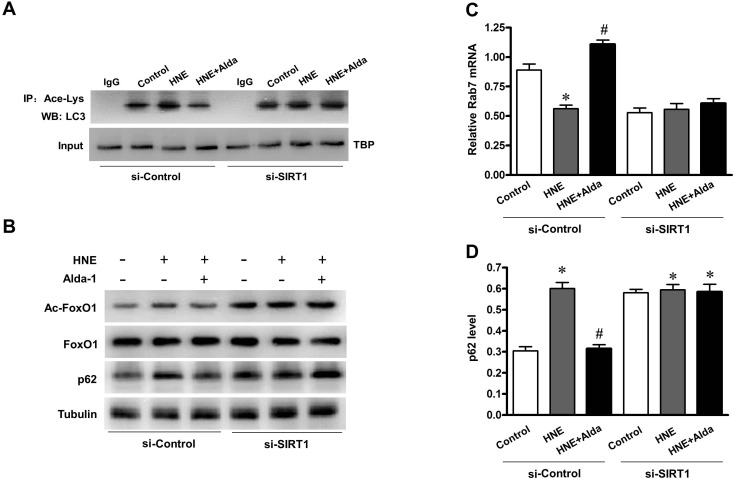
SIRT1 is essential for ALDH2-ameliorated autophagic flux under carbonyl stress

To evaluate whether SIRT1 is required for ALDH2-induced autophagy, Ad-sh-Sirt1-transduced myocytes were subjected to carbonyl stress with or without Alda-1 treatment. SIRT1 protein was significantly decreased in myocytes after transfection with Ad-sh-Sirt1. Knockdown of SIRT1 abolished the ALDH2-induced deacetylation of LC3 and FoxO1 in myocytes (Figure 9A, 9B). In line with this, Rab7 expression was also increased significantly on ALDH2 activation, but this increase was revoked when SIRT1 was knockdown by Ad-sh-Sirt1 (Figure 9C). Furthermore, the ALDH2-induced p62 degradation was significantly attenuated on knockdown of SIRT1 (Figure 9D). These results suggest that SIRT1 is required for ALDH2 activation induced autophagy in myocytes against carbonyl stress.

Cardiomyocytes were transduced with Ad-sh-Sirt1 (96 h) followed by 1 hour aldehydic overload (4-HNE, 40 μmol/L) in the absence or presence of Alda-1 treatment (20 μmol/L). **A.** Acetylation of LC3 in nuclear fractions was assessed. **B.** Western blot analysis of acetylated FoxO1 (Ac-FoxO1), total FoxO1 and p62 proteins. **C.** Quantificational detection showing Rab7 mRNA level. **D.** Densitometric analyses showing p62 protein level (*n* = 4 per group, *P < 0.05 *vs*. normal control; #*P* < 0.05 *vs*. 4-HNE alone).

## DISCUSSION

Cardiac aging is characterized by impaired local protein environments and proteostasis, including decreased protein turnover and/or increased proteotoxicity [15]. Myocardium, as postmitotic tissues, is particularly susceptible to proteotoxicity because sustained and severe proteotoxic stress leads to cell death and the cardiomyocyte has very limited self-renewal capacity [16]. Age-associated proteostasis disorders in cardiovascular (CV) structure or function below “clinical threshold” ought not to be considered to reflect “physiologic” CV aging. Rather, in the context of “subclinical” disorder, age-associated changes might be construed as specific risk factors for the CV diseases that they relate to and thus might be targets of interventions for decrease or delay of the occurrence or severely reduce cardiovascular disease at later ages. Here, we identify a link between ALDH2 function and the cardiac aging process and show that the age-associated ALDH2 deficiency results in aldehydic overload and carbonyl damage in hearts, which triggers inactivation of key proteins (i.e. SIRT1). In turn, impaired SIRT1 activation contributes to the secondary impairment of autophagy that, by negatively affecting deacetylation of LC3 and FoxO1, ensues in further accumulation of proteotoxicity. We believe that ALDH2 dysfunction lies at the critical point of a vicious cycle in aging process. Disrupting this vicious linkage by reducing cardiac aldehydic load through selective ALDH2 activation decreases carbonyl stress and enhances autophagy, therefore slowing down the aging process and contributing to better health.

Much of what we knew about ALDH2 is traceable to ethanol metabolism. Nevertheless, an exciting body of more recent research indicates that ALDH2 has emerged as a key enzyme in cardioprotection, since it efficiently eliminates toxic aldehydes [3]. Decrease in detoxification of lipid peroxidative aldehydes has been observed and proposed as an underlying mechanism of aging in animals. Recent studies suggest that ALDH2 dysfunction is associated with the process of aging. Also, we previously demonstrated that pharmacological activation of ALDH2 protects the aged heart against acute ischemia/reperfusion injury [5, 17]. However, the major factor and mechanistic link between ALDH2 activity and aging-associated damage remain as yet poorly answered. Are therapeutic agents that activate ALDH2 beneficial in reducing aging-induced cardiac damage? Using young and aged mice model, we found that myocardial ALDH2 activity decreased in aged hearts compared with that in young control. Meanwhile, our data depicted a pronounced increase in aldehydic load in aged hearts, characterized by accumulation of cardiac 4-HNE protein adducts and protein carbonyls. Our functional and biochemical analyses at different ages demonstrated that ALDH2 ablation mice accelerated cardiac aging progress, which has shortened their lifespan. 4-HNE can readily interact with cysteine, histidine, and lysine residues and inactivate key proteins *via* carbonyl modification [18]. 4-HNE has been shown to be a substrate as well as a potent inhibitor of ALDH2 (due to 4-HNE adduct formation on ALDH2). Substrate-induced inactivation may be a possible mechanism for the ALDH2 inactivation in aging myocardium. We demonstrated that cardiac SIRT1 was the carbonylation target by aldehyde stress which consequently resulted in SIRT1 inactivation. Moreover, selective ALDH2 activation attenuated cardiac aldehydic load and the level of carbonylated SIRT1 seen in aged hearts. We showed that sustained treatment with Alda-1 is well tolerated in aged mice, suggesting that chronic activation of ALDH2 is safe.

When cardiac protein damage occurs, autophagy must intervene. Continuous constitutive autophagy has a crucial role in maintaining cardiac function during aging by controlling the quality of proteins and mitochondria [19]. In aged and ALDH2-null hearts, the evidence we have collected clearly shows that the accumulation of autophagosomes was confirmed by LC3 immunofluorescence staining and autophagic flux was impaired as indicated by increased p62 protein levels. Characterization of autophagic flux by assessing autophagosome clearance mediated by lysosomes demonstrated that autophagosome accumulation was caused by the impairment of autophagosome removal. Thus, ALDH2 disruption causes accumulation of autophagosomes *via* impairment of autophagic flux *in vivo* in aged hearts. Interestingly, selective ALDH2 activation restored the autophagic flux in aged hearts but not Sirt1^+/−^ hearts, indicating that SIRT1 is required for ALDH2 activation induced autophagy. Starvation of newborn mice lacking atg5 increases perinatal death resulting from heart failure, suggesting that the heart function critically relies on autophagy during nutrient starvation [20]. Consistent with a requirement for SIRT1 in the execution of ALDH2-induced autophagy, a decrease in cardiac function caused by starvation was also observed in Sirt1^+/−^ hearts even in the presence of ALDH2 activation.

We found that ALDH2 activation could protect SIRT1 activity and promote nuclear pool of SIRT1. Protein acetylation is known to play an important role in autophagy [21]. We next sought to characterize further the role of SIRT1 in regulating acetylation of the proteins controlling autophagy in aged hearts *via* selective ALDH2 activation. We thought it possible that nuclear SIRT1 might interact directly with components of the autophagy machinery. Experiments with Ad-tf-LC3 allowed us to demonstrate that ALDH2 activation not only stimulate autophagosome but also enhance formation of autolysosomes, and, thus, they both stimulate myocardial autophagic flux under carbonyl stress. Nuclear LC3 plays important role in the formation of autophagosomes. Most recent *in vitro* cell lines studies have demonstrated that deacetylation of nuclear LC3 by SIRT1 drives autophagy initiation [12]. Whether nuclear LC3 participates in the ALDH2 induced autophagy in aged heart *in vivo* is unclear. Indeed, as mentioned, we confirmed that deacetylation of LC3 in nucleus by SIRT1 was increased in aged hearts following Alda-1 treatment and subsequent enhanced LC3-Atg7 interaction in cytoplasmic pools. For another, impaired autophagic flux can occur from functional disorder of lysosome-autophagosome fusion. Rab7, a small GTPase protein, plays an important role in the fusion of the matured autophagic vacuole with the lysosome. Previous studies have indicated that FoxO1 regulates expression of Rab7 and SIRT1 is an upstream regulator of FoxO1. However, whether or not the effect of ALDH2-activated SIRT1 on autophagy is mediated through FoxO1-Rab7 signal is unknown. In this study, our *in vivo* data shown that Alda-1 treatment enhanced Sirt1-mediated deacetylation of FoxO1 and upregulation of Rab7 in aged hearts. Finally, knockdown of SIRT1 abolished ALDH2 activation against carbonyl stress induced deacetylation of nuclear LC3 and FoxO1 in myocytes. In this report, our data would suggest that by preventing carbonylation, ALDH2-activated SIRT1 may play a pivotal role in regulating the multi-step of autophagy flux. Given the central role of proteostasis in the aging process, the role of ALDH2 in inhibition of protein carbonyl damage and potentially rejuvenating the autophagy flux may provide important insight into the overall cardioprotection of ALDH2 function.

In conclusion, our *in vivo* findings highlight that loss of ALDH2 per se is sufficient to cause cardiac damage, which evolves into impaired proteostasis when protein quality control becomes less efficient, as occurs in aging. Genetic polymorphisms of human ALDH2 have been well surveyed. Further studies will aim to determine the potential impact on senescence of an inactivating single point mutation in ALDH2 that is found in ~560 million East Asians (ALDH2*2 mutation) [4, 22], or nearly 8% of the world's population.

## MATERIALS AND METHODS

### Animals

Male C57BL/6 mice (4 and 22 mo) were purchased from the animal center of Fourth Military Medical University. Male ALDH2 knockout mice (ALDH2 KO, C57BL/6) were gifts from Dr. H. Zhang (Fuwai Hospital, National Center for Cardiovascular Disease, China). ALDH2 KO mice were backcrossed into the C57BL/6 background (generation N10) at the animal resource center. SIRT1 deficient heterozygous (Sirt1^+/−^) mice (3-4 mo) were obtained from The Jackson Laboratory (Bar Harbor, ME). Sirt1^+/−^ mice were backcrossed with C57BL/6 mice for 6 generations to obtain the C57BL/6 gene background. The experiments were performed in adherence with the National Institutes of Health Guidelines on the Use of Laboratory Animals and were approved by the Fourth Military Medical University Committee on Animal Care.

### Treatment with Alda-1

Aged mice were treated with Alda-1 (selective ALDH2 activator; Calbiochem #126920) for 4 weeks. Alda-1 dose (3 mg/kg BW/per injection, dissolved in 50% polyethylene glycol and 50% dimethyl sulfoxide by volume) was established in preliminary studies and given in multiple doses (3 doses per day) [22]. Alda-1 was injected subcutaneously to the dorsal side of the neck. One subset of aged animals received the vehicle alone served as the control.

### ALDH2 enzymatic activity

ALDH2 enzymatic activity was determined by measuring the conversion of NAD^+^ to NADH at absorbance of 340 nm, as described [5, 6, 23, 24]. ALDH2 activity was measured at 25°C in 33 mmol/L sodium pyrophosphate containing 0.8 mmol/L NAD^+^, 15μmol/L propionaldehyde, and 0.1 ml protein extract (50 μg of protein). Propionaldehyde, the substrate of ALDH2, was oxidized in propionic acid, whereas NAD+ was reduced to NADH to estimate ALDH2 activity. NADH was determined by spectrophotometric absorbance at 340 nm. ALDH2 activity was expressed as nmol NADH/min per mg protein.

### Total protein and SIRT1 carbonylation assessment

The protein carbonyl content of tissue was determined as described previously [5, 14, 25]. The carbonyl groups in the protein side chains were derivatized to 2,4-dinitrophenylhydrazone (DNPhydrazone) by reaction with 2,4-dinitro phenylhydrazine (DNPH). The DNP-derivatized protein samples were separated by polyacrylamide gel electrophoresis followed by immunoblotting. Additional studies were performed to detect covalent modification of SIRT1 by carbonylation. SIRT1 was immunoprecipitated using whole-cell extracts in accordance with published methods [26]. To determine the carbonylation of SIRT1, blots were probed first with anti-SIRT1 antibody. After stripping, membranes were equilibrated with 20% (v/v) methanol, 80% Tris-buffered saline for 5 min. Then they were incubated with 0.5 mmol/L 2,4-DNPH for 30 min at room temperature. The membranes were washed and then incubated overnight in anti-DNPH antibody, as described previously [5].

### SIRT1 activity assay

SIRT1 deacetylase activity was evaluated in crude nuclear extract from heart samples [5]. Trichostain A (0.2 mM; Sigma-Aldrich, St. Louis, MO, USA), components of Fluor de Lys SIRT1 Fluorescent Activity Assay/Drug Discovery Kit (Enzo Life Sciences, Farmingdale, NY, USA), including 100 μmol/L fluorogenic peptide encompassing residues 379 to 382 of p53 with lysine 382 being acetylated, and 170 μmol/L NAD+ at 37°C for 1 h, followed by incubation in developer for 15 min at room temperature according to the manufacturer's instructions. Fluorescent intensity was measured using a Fluoroskan Ascent^®^ microplate fluorometer (Thermo Electron Corp., Milford, MA, USA). No-enzyme and time 0 negative controls were generated by incubating developer II solution with 2 mmol/L NAM before mixing the substrates with or without samples. SIRT1 activity was calculated with the corrected arbitrary fluorescence units of the tested samples to noenzyme control and expressed as fluorescent units relative to the control. The nuclear-cytoplasmic fraction of heart tissue was conducted using the NE-PER Nuclear and Cytoplasmic Extraction Reagents kit (Thermo, Fisher Scientific, Rockford, IL, USA) [26].

### Echocardiography

Mice were anesthetized using 12 μL/g BW of 2.5% avertin (Sigma-Aldrich), and cardiac function was determined using echocardiography (VisualSonics VeVo 770) as previously described [27].

### Western blotting analysis and Immunoprecipitation

Immunoblots were performed as previously described [5]. Antibodies against p16 (1:200 dilution; #sc-1207), ALDH2 (1:200 dilution; #sc-100496) and acetylated FoxO1 (1:200 dilution; #sc-49437) were purchased from Santa Cruz Biotechnology (Santa Cruz, CA, USA). Antibodies against p53 (1:1000 dilution; #2524), LC3B (1:1000 dilution; #3868), SirT1 (1:1000 dilution; #3931), acetyl-lysine (1:1000 dilution; #9441), Atg7 (1:1000 dilution; #8558), Rab7 (1:1000 dilution; #9367) and FoxO1 (1:1000 dilution; #2880) were purchased from Cell Signaling Technology. Antibody against DNPH (1:2000 dilution; #ab93160), LAMP2 (1:1000 dilution; #ab25339), Tubulin (1:1000 dilution; #ab179513), GAPDH (1:10000 dilution; #ab181602) and TBP (1:5000 dilution; #ab28175) were purchased from Abcam. Antibody binding was detected *via* enhanced chemiluminescence (Millipore) and scanned with ChemiDocXRS (Bio-Rad Laboratory, Hercules, CA). Immunoblot band intensity was analyzed with Lab Image software. For immunoprecipitation analysis, lystes were mixed with primary antibody. Immunocomplexes were separated by SDS-PAGE and detected with western blot analyses.

### Bafilomycin mouse treatment

Bafilomycin A1, a membrane-permeant lysosomal inhibitor, is a vacuolar H+-ATPase inhibitor that inhibits autophagosome-lysosome fusion to prevent the final digestion step of autophagy. Bafilomycin (Sigma-Aldrich, #1793) was dissolved in DMSO and intraperitoneally injected in young and aged mice at a dosage of 0.3 mg/kg. Treatments were administered by daily intraperitoneal injection for 1 week. The dose of bafilomycin used here was previously reported not to cause apparent adverse effects and successfully suppress autophagy in the mouse heart. A group of control mice injected with the same amount of the vehicle was also tested, as described previously [28].

### Adenoviral transduction

The method to culture cardiac myocytes has been described previously[10]. Transductions with Ad-tf-LC3 and Ad-GFP-LC3 (Hanbio Inc, China) were carried out for 24 hours. Knockdown adenoviruse of Ad-sh-Sirt1 was transduced for 96 hours. Adenoviruses were transduced at 50 MOI [10].

### Fluorescence microscopy

The method to evaluate tandem fluorescent LC3 puncta using Ad-tf-LC3 has been described previously [10]. Cardiac myocytes cultured on cover slips were transduced with adenovirus harboring tandem fluorescent mRFP-GFP-LC3 (Ad-tf-LC3) at 50 MOI. 24 hours after adenovirus transduction, the cells were washed and fixed with 4% paraformaldehyde, mounted with a reagent containing 4′,6-diamidino-2-phenylindole (DAPI) (Vectashield, Vector Laboratories Inc.), and viewed under a fluorescence microscope (Nikon Eclipse E800). The number of GFP and mRFP dots was determined by manual counting of fluorescent puncta from at least 4 different myocyte preparations with a 60× objective. At least 50 cells were scored in each experiment. The nuclear number was evaluated by counting the number of DAPI stained nuclei in the same field. The number of dots/cell was obtained by dividing the total number of dots by the number of nuclei in each microscopic field.

### Statistical analysis

Differences between 2 groups were compared with Student *t*-test. For groups more than 2, differences were compared by analysis of variance (ANOVA) followed by the Scheffe's correction for post-hoc *t*-test comparison. Probabilities of 0.05 or less were considered statistically significant.

## SUPPLEMENTARY FIGURES AND TABLES


