# Supplementary material for: Aldehyde dehydrogenase 2 activation in aged heart improves the autophagy by reducing the carbonyl modification on SIRT1
Source: Oncotarget. 2016 Jan 3;7(3):2175–88. doi: 10.18632/oncotarget.6814 (PMC4823027; doi:10.18632/oncotarget.6814)
Supplement: Supplementary file 1 [file oncotarget-07-2175-s001.pdf]

Aldehyde dehydrogenase 2 activation in aged heart improves the autophagy by reducing the carbonyl modification on SIRT1

Supplementary Material

Supplementary Table 1

General features of young (4 mo) and aged (22 mo) mice

Mouse group	Young	Aged
**Body weight(BW, g)**	24.2 ± 1.5	32.3 ± 0.9*
**Heart weight (HW, mg)**	140 ± 4	165 ± 6*
**HW/BW (mg/g)**	5.78 ± 0.26	5.11 ± 0.66
**Normalized LV mass (mg/g)**	2.17±0.5	2.25±0.3
**Fractional shortening (%)**	48.6±1.46	46.5±3.4
**Heart rate (beats.min^{-1})**	465±12	458±32

Values are means ± SEM, n=10 for both groups, **P*<0.05 vs Young

Supplementary Table 2

General features of WT and ALDH2 KO mice

Mouse group	WT	ALDH2 KO
**Body weight (g)**	25.8 ± 1.2	23.5 ± 1.5
**Heart weight (mg)**	145± 9	143± 11
**Heart weight/body weight ratio (mg/g)**	5.62± 0.51	6.08 ± 0.42
**Liver weight (g)**	1.35±0.03	1.32±0.05
**Liver weight/body weight ratio (mg/g)**	51.2±2.50	56.2±3.33
**Kidney weight (mg)**	355±8	338±12
**Kidney weight/body weight ratio (mg/g)**	13.75±0.67	14.38±0.80
**Fractional shortening (%)**	47.6±1.5	42.3±2.3
**Heart Rate (beats.min^{-1})**	452±18	460±30

Values are means ± SEM, n=10 for each groups
